# Decreasing Total Medication Exposure and Length of Stay While Completing Withdrawal for Neonatal Abstinence Syndrome during the Neonatal Hospital Stay

**DOI:** 10.3389/fped.2017.00216

**Published:** 2017-10-10

**Authors:** Lori A. Devlin, Timothy Lau, Paula G. Radmacher

**Affiliations:** ^1^Division of Neonatal Medicine, Department of Pediatrics, University of Louisville School of Medicine, Louisville, KY, United States; ^2^Department of Educational and Counseling Psychology, University of Louisville, Louisville, KY, United States

**Keywords:** neonatal abstinence syndrome, length of stay, neonatal, drug withdrawal, protocol, infant, newborn

## Abstract

**Introduction:**

Neonatal abstinence syndrome (NAS) is a rapidly growing public health concern that has considerably increased health-care utilization and health-care costs. In an effort to curtail costs, attempts have been made to complete withdrawal as an outpatient. Outpatient therapy has been shown to prolong exposure to medications, which may negatively impact neurodevelopmental and behavioral outcomes. We hypothesized that the implementation of a modified NAS protocol would decrease total drug exposure and length of stay while allowing for complete acute drug withdrawal during the neonatal hospital stay.

**Methods:**

Data were derived retrospectively from medical records of term (≥37 0/7) infants with NAS who were treated with pharmacologic therapy in the University of Louisville Hospital Neonatal Intensive Care Unit from 2005 to 2015. The pharmacologic protocol (SP1) for infants treated between 2005 and March 2014 (*n* = 146) dosed oral morphine every 4 h and utilized phenobarbital as adjuvant therapy. Protocol 2 (SP2) initiated after March 2014 (*n* = 44) dosed morphine every 3 h and used clonidine as adjuvant therapy. Charts were reviewed for demographic information and maternal drug history. Maternal and infant toxicology screens were recorded. The length of morphine therapy and need for adjuvant drug therapy were noted. Length of stay was derived from admission and discharge dates.

**Results:**

The length of morphine therapy was decreased by 8.5 days from 35 to 26.5 days (95% CI 4.5–12 days) for infants treated with SP2 vs. SP1 (*p* < 0.001). The need for adjuvant pharmacologic therapy was decreased by 24% in patients treated with SP2 vs. SP1 (*p* = 0.004). The length of stay was decreased by 9 days from 42 to 33 days (95% CI 5.1–13 days) for infants treated with SP2 vs. SP1 (*p* < 0.001). The decreased length of stay resulted in an average reduction of hospital charges by $27,090 per patient in adjusted 2015 US Dollars.

**Conclusion:**

This study demonstrates that total drug exposure and length of stay can be reduced while successfully completing acute withdrawal during the neonatal hospital stay.

## Introduction

Perinatal opiate abuse is a rapidly expanding public health concern that has markedly increased health-care costs and utilization. Neonatal abstinence syndrome (NAS) occurs in the exposed infant after the abrupt cessation of maternal opiates at birth and accounts for a substantial proportion of the health-care burden (1, 2). The national incidence of NAS increased from 3.4 to 5.8 per 1,000 hospital births between 2009 and 2012; Neonatal Intensive Care Unit (NICU) admissions for NAS increased fourfold between 2004 and 2013 (1, 3). Regional and state variation in the incidence of NAS has been found to cause disproportionate burden on highly affected areas such as the southeastern United States (3, 4).

Clinically significant symptoms appear in 55–94% of opiate exposed neonates (5). The onset and severity of symptoms are impacted by the sum total of fetal exposure, the type(s) and purity of drug(s) consumed during pregnancy, maternal and infant metabolism, genetic and epigenetic factors, and variability in the kinetics of placental drug transfer (6–11). Infants who require pharmacologic therapy to treat withdrawal symptoms are cared for in the NICU in many regions of the US (1). The percentage of NICU days attributed to NAS increased from 0.6% in 2004 to 4% in 2013 (1). The proportion of NAS infants who require pharmacotherapy has also increased from 74% in 2004–2005 to 87% in 2012–2013 (1). The severity of withdrawal is assessed with observer-rated scales. The most commonly used scale is the Finnegan Score for Neonatal Abstinence Syndrome (FNAS) (5). FNAS assesses 21 clinical symptoms of drug withdrawal, which allows for a thorough evaluation (6). However, it is a lengthy tool that requires extensive training and experience to ensure accurate scoring (12). Variability in the application of FNAS complicates care and prolongs hospitalization for infants with NAS (13–17).

The surge in the number of infants diagnosed with NAS, variability in the assessment of symptoms and increased need for pharmacologic treatment of affected infants has intensified the financial impact of the disease. National aggregate hospital charges reached $1.5 billion in 2012 (3). State Medicaid programs are financially responsible for 80% of the cost for the treatment of NAS (3). Implementation of measures to stunt the growing economic impact of NAS while ensuring the short- and long-term safety and development of affected infants is crucial. Primary prevention measures could markedly decrease the incidence of NAS (4). Unfortunately, such measures require diligence and compliance from the dependent mother in conjunction with her health-care providers, which are often difficult to attain. Secondary interventions including pharmacologic and non-pharmacologic therapy for the withdrawing neonate remain variable and best practice has yet to be established (13, 18).

In 2014, a group of neonatologists (including the primary author), nurse practitioners, and neonatal nurses from the regional referral centers in Kentucky were tasked with standardizing pharmacologic treatment for affected infants throughout the state. Subsequently, a new treatment protocol was developed based on the best-available evidence. In Kentucky, infants requiring pharmacologic care for acute opioid withdrawal are typically cared for in an NICU. We hypothesized that implementation of the state-vetted protocol would decrease postnatal drug therapy by decreasing the total days of morphine therapy and the need for adjuvant therapy. In addition, we expected that a decrease in pharmacologic therapy would improve the length of stay and reduce hospital costs. All infants evaluated in this study were treated in the NICU of a single inner-city hospital between 2005 and 2015, and all infants completed pharmacologic therapy before discharge.

## Materials and Methods

The University of Louisville Institutional Review Board approved this retrospective cohort study before study initiation. Data were derived from medical records of infants treated in the University of Louisville Hospital Neonatal Intensive Care Unit (Louisville, KY, USA) from 2005 to 2015. NICU log books were used to identify term infants (greater than or equal to 37.0/7 weeks gestation) with NAS. Charts were reviewed for demographic information including sex, gestational age, and birth weight. Maternal drug history and maternal and infant toxicology screens were recorded (Roche Cobas Integra 800). Pharmacologic intervention, including the length of morphine therapy and need for adjuvant drug therapy, was obtained. Length of stay was derived from admission and discharge dates for each infant.

**Figure 1:**
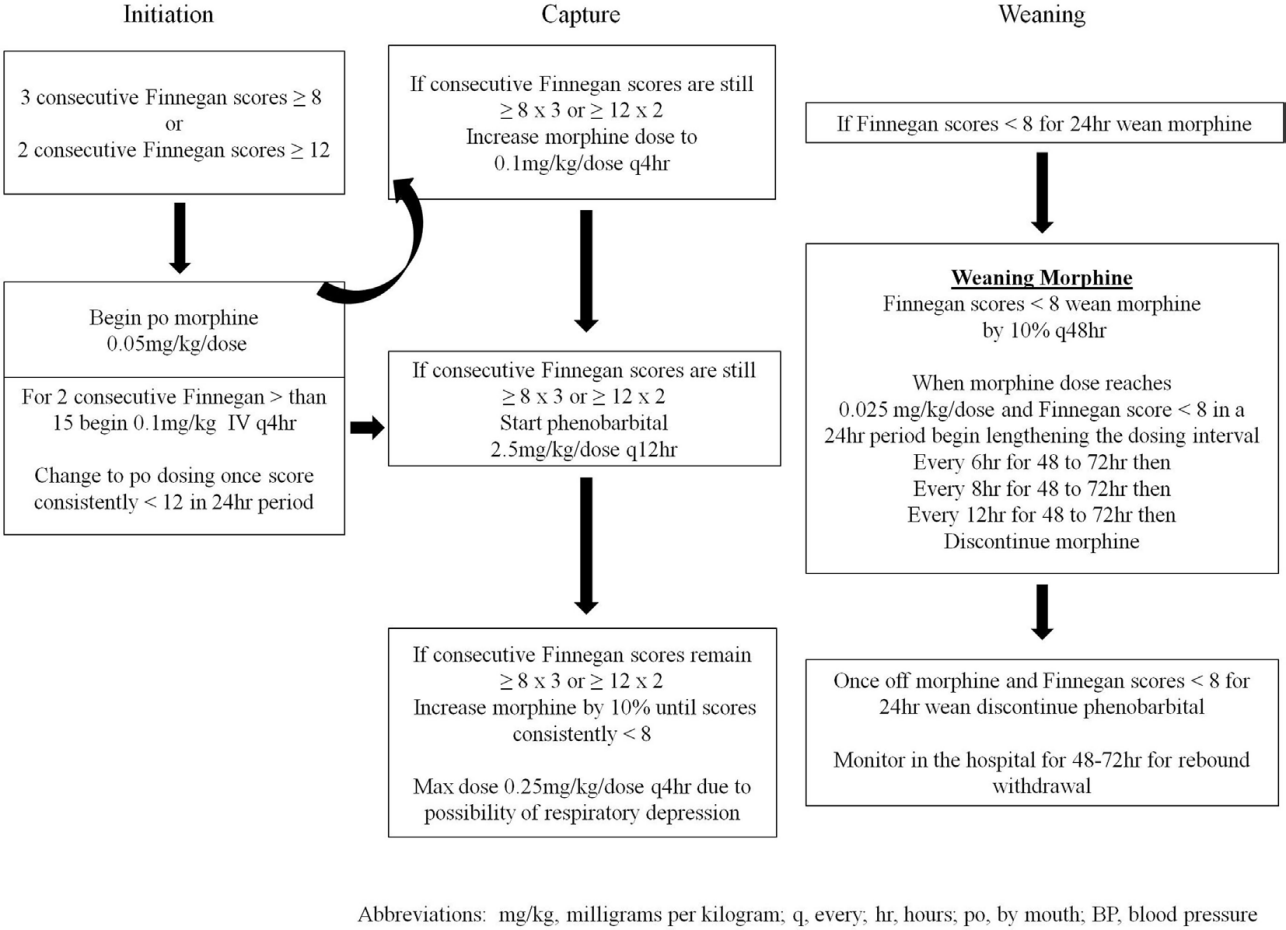
Protocol 1 (SP1) for the initiation and treatment of neonatal abstinence syndrome.

**Figure 2:**
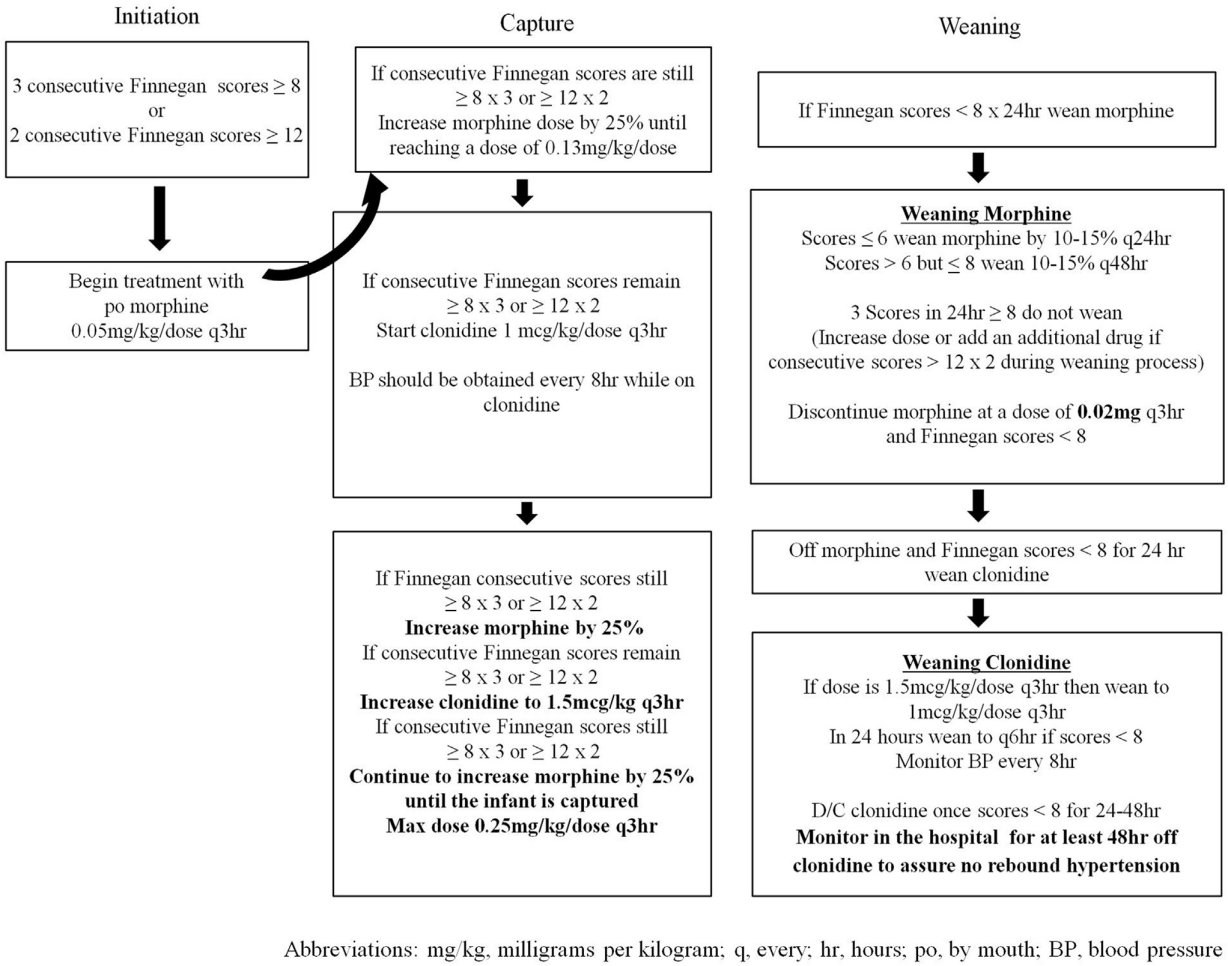
Protocol 2 (SP2) for the initiation and treatment of neonatal abstinence syndrome.

Infants cared for in the NICU between 2005 and March 2014 (*n* = 146) were treated with a locally generated protocol (SP1—Figure 1), and infants treated after March 2014 (*n* = 44) were treated with the state-generated protocol (SP2—Figure 2). Both treatment protocols used morphine as the first pharmacologic agent; however, morphine dosing was every 4 h in SP1 and every 3 h in SP2. Weaning of the morphine was also more aggressive in SP2 vs. SP1. In addition, infants treated with SP1 received phenobarbital as adjuvant therapy if symptoms were not well controlled, on high dose oral morphine while infants treated with SP2 received clonidine.

Differences in birth weight and gestational age between treatment groups were assessed with a Student’s *t*-test, and differences in sex between groups were assessed with a χ^2^ test (goodness of fit) SPSS version 22. ANCOVA (R version 3.1.2) was used to evaluate the impact of each protocol on the length of morphine therapy and length of hospital stay. The model controlled for birth weight, gestational age, sex, and fetal exposure to heroin, methadone, buprenorphine, multiple opiates, and benzodiazepines. Exposure was determined by maternal history and/or maternal and infant toxicology screens. Interactions between *in utero* drug exposure and the protocol were evaluated with backwards elimination multiple regression. The need for adjunctive therapy in the infants treated with each protocol was assessed with a with a χ^2^ test (goodness of fit) SPSS version 22. For all analyses, statistical significance was set at *p* < 0.05.

## Results

The primary outcome measure was length of morphine therapy. Secondary outcomes were need for adjunctive therapy and length of stay. Demographic data are presented in Table 1. No statistically significant differences were noted in the gestational age, weight, or sex between infants treated with SP1 or SP2.

**Table 1:** Demographics of study infants.

	Protocol 1 (SP1) (*n* = 146)	Protocol 2 (SP2) (*n* = 44)	*p*-Value
Birth weight (kg)	3.01 ± 0.46	2.93 ± 0.39	0.07^a^
Gestational age (weeks)	39.0 ± 1.1	38.7 ± 1.3	0.21^a^
Male (%)	57.2	59.1	0.82^b^

*^a^p-Values calculated with t-test*.

*^b^p-Value calculated with χ^2^ test of independence*.

**Figure 3:**
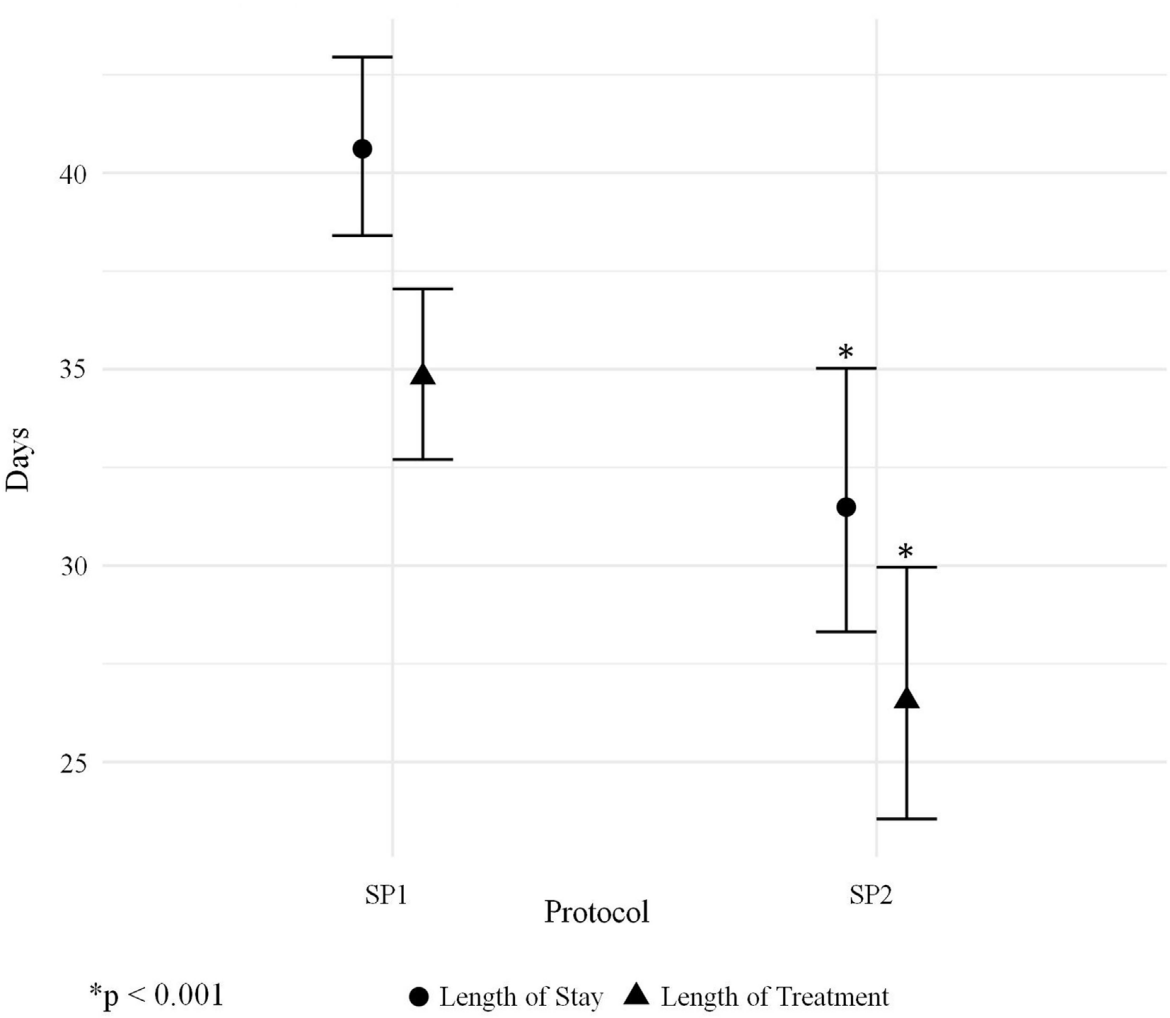
Adjusted means for length of morphine therapy and length of stay.

**Figure 4:**
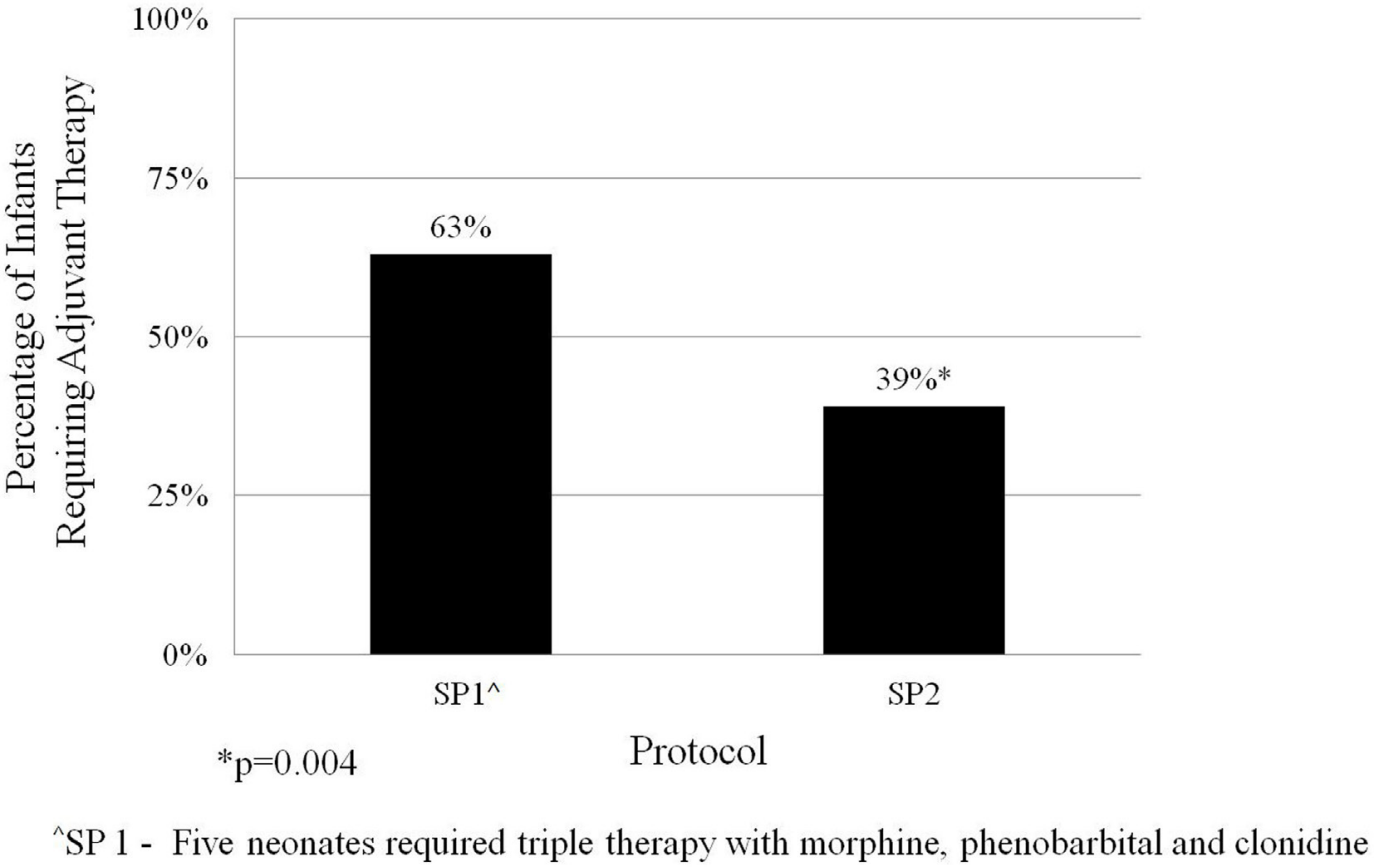
Adjuvant therapy for neonatal abstinence syndrome by protocol.

The average length of morphine therapy was decreased for infants treated with SP2 by 8.5 days from 35 to 26.5 days (95% CI 4.5–12 days) when compared with infants treated with SP1 (*p* < 0.001) (Figure 3). The need for adjunctive pharmacologic therapy was decreased by 24% in patients treated with SP2 vs. SP1 (*p* = 0.004) (Figure 4). The average length of stay for infants treated with SP2 was decreased by 9 days from 42 to 33 days (95% CI 5.1–13 days) when compared with infants treated with SP1 (*p* < 0.001) (Figure 3). The decrease in length of stay was calculated to result in an average reduction of hospital charges by $27,090 per patient (adjusted 2015 US Dollars) using national averages for per day charges derived by Patrick et al. (3). The use of multiple opiates and benzodiazepines was not found to be statistically different between protocols (Table 2).

**Table 2:** Fetal drug exposure to opioids and other drugs of abuse.^a^

	Total	Protocol 1 (SP1)	Protocol 2 (SP2)
	Number (%) (*n* = 190)	Number (%) (*n* = 146)	Number (%) (*n* = 44)
**Opioids**			

Buprenorphine	18 (9)	9 (6)	9 (20)
Heroin	24 (12)	13 (8)	11 (25)
Hydrocodone	29 (15)	28 (19)	4 (9)
Hydromorphone	4 (2)	2 (1)	2 (5)
Methadone	74 (39)	61 (42)	13 (30)
Morphine	11 (6)	3 (2)	8 (18)
Oxycodone	47 (24)	43 (29)	4 (9)
Oxymorphone	5 (3)	4 (3)	1 (2)
Unspecified opiates	88 (46)	67 (46)	21 (48)

**Other drugs of abuse**			

Amphetamines	11 (6)	8 (5)	3 (7)
Barbiturates	2 (1)	2 (1)	0
Benzodiazepines	20 (10)	18 (10)	2 (5)
Cocaine	26 (14)	19 (13)	7 (15)
Gabapentin	2 (1)	2 (1)	0
Marijuana	18 (9)	14 (10)	4 (9)
Methamphetamine	4 (2)	2 (1)	2 (5)

*^a^Fetal exposure was determined through maternal history, and urine toxicology screens combined with neonatal urine and meconium toxicology screens*.

One relevant and significant interaction was noted within the backwards elimination multiple regression between methadone and protocol 2 (SP2). While the average difference in length of stay for all infants was 8.5 days when comparing SP2 vs. SP1, infants exposed to methadone *in utero* had a 1-day reduction in the benefit (e.g., an average difference of 7.5 vs. 8.5 days).

## Discussion

In this cohort of infants from a single inner-city hospital, we found that medication exposure and length of stay were decreased with the implementation of a state endorsed standardized pharmacologic treatment protocol. This finding lends additional support to the efficacy and cost effectiveness of standardized treatment for infants with NAS (5, 19–23). It also highlights the importance of statewide collaborative efforts in enhancing short-term outcomes and reducing hospital costs.

All of the infants in our cohort were exposed *in utero* to opioids; the type(s) of exposure was variable as depicted in Table 2. Total fetal exposure was wide ranging and difficult to quantify due to maternal misreporting and limitations in maternal and infant toxicology screens. Fetal exposure to multiple opiates and multiple drugs of abuse, specifically benzodiazepines and tobacco, is known to exacerbate the severity of withdrawal (24). In our data analyses, we controlled for exposure to multiple opiates and benzodiazepines and did not find a statistically significant impact on the effectiveness of the protocol. Previous studies have shown that infants exposed to methadone require a longer length of treatment than those exposed to buprenorphine and other short acting opiates such as heroin (14). We found that infants exposed to methadone *in utero* and then treated with SP2 vs. SP1 had an average length of morphine therapy and length of stay that was 1 day greater than infants exposed to other opiates during pregnancy (*p* = 0.003). In other words, infants exposed to methadone do not receive as much benefit from the new protocol as infants not exposed to methadone. Additional research is needed to evaluate the efficacy of tailoring treatment protocols to *in utero* exposure.

Prenatal exposure to opiates, especially long acting opiates such as methadone, has been shown to decrease myelination in the developing rat brain and in a small cohort of exposed infants (25, 26). The endogenous opioid system is crucial to ensure appropriate oligodendrocyte development and proper myelination. *In utero* exposure to exogenous opiates inhibits endogenous opioid production and may decrease myelination in the brain (26). *In utero* opioid exposure has also been correlated with decreased brain volumes and suboptimal long-term developmental outcomes (25, 27, 28). Little is known about the specific impact of postnatal opioids on the developing brain. Given the negative neurological outcomes of intrauterine exposure, every effort should be made to decrease the duration of postnatal opioid treatment. In our study infants treated with SP2 had an average of 8.5 fewer days of postnatal opiate therapy than infants treated with SP1. Although the impact was modest, we were able to demonstrate a decrease in total postnatal opioid exposure.

Adjunctive therapy has been used to decrease the duration of opioid treatment during acute neonatal withdrawal. Clonidine and phenobarbital are the most commonly used agents. Phenobarbital has been noted to decrease the severity of opioid withdrawal and reduce length of stay and cost of hospitalization (19, 29, 30). Of note, infants enrolled in these studies were discharged home on phenobarbital. Many infants remained on the medication for weeks to months after the initial hospital discharge (19, 24, 29). Phenobarbital acts on the gamma-aminobutyric acid receptors in the central nervous system inducing sedative effects. Extended treatment with phenobarbital may negatively impact neurodevelopmental and behavioral outcomes (31). Clonidine has been evaluated in several small studies and has been shown to be a safe and efficacious adjunctive therapy for infants with NAS. The use of clonidine in the pharmacologic management of infants undergoing acute opiate withdrawal has been associated with a decrease in the duration of treatment and length of stay (32–35). Yet, it should be noted that the impact of clonidine on developmental outcomes remains unknown. In view of the uncertain long-term impact of both commonly used adjuvant medications, treatment with a single opioid would likely prevent potential side effects and decrease the chance of harm. We found that simply transitioning morphine from every 4 to every 3 h led to a 24% reduction in the need for adjuvant therapy.

The rapid growth of *in utero* opiate exposure and NAS over the last decade has stretched health-care resources and escalated health-care costs. Between 2000 and 2009, national estimates for total hospital charges attributed to NAS increased from $190 million to $720 million (2). Inflation-adjusted mean hospital charges for infants requiring pharmacologic therapy for NAS in 2012 reached $93,400 per patient (3). State Medicaid programs bear the majority of the financial burden and marked variability exists between states (4). Using data from Patrick et al (3), a mean extrapolated daily charge for NAS pharmacologic treatment is approximately $3,010.00 (2015 adjusted dollars). When extrapolated costs were applied to our study population, the use of SP2 decreased hospital charges by $27,090 per patient. When applied to the 44 patients treated with SP2, the average decrease in hospital charges approached $1.19 million. Prolongation of the hospital stay due to child protective services (CPS) interventions may have affected the differences in length of stay. We did not collect data on incidence of CPS engagement and thus cannot determine the effect of this parameter on our outcomes.

In this study, the implementation of a pharmacologic treatment protocol developed by a statewide collaboration led to a decrease in total opioid exposure, need for adjunctive therapy, and length of stay while completing acute withdrawal during the neonatal hospitalization. Completing pharmacologic therapy before initial hospital discharge will decrease total medication exposure and has the potential to decrease harm in a population that is known to be at increased risk for adverse post-discharge outcomes (32, 36).

The results of this study focus exclusively on the impact of modifying the pharmacologic treatment protocol. It is well known that the severity of opioid withdrawal is dependent upon and affected by a multiplicity of factors. The impact of environment, breastfeeding, and parental engagement cannot be under estimated (33–35, 37–40). The development of a comprehensive evidence-based treatment protocol will require consideration of all impacting factors. We acknowledge that the study is limited in its retrospective design and thus is subject to bias and inaccuracies within the medical record. We also realize that only the impact of the entire treatment protocol can be assessed with this study design. In addition, patients treated with SP1 were collected over a 9-year period during which trends in maternal usage and drugs of abuse may have varied from current patterns of abuse during pregnancy. Subtle changes in neonatal care over the last 11 years could have impacted on the short-term outcomes for affected infants.

## Author Contributions

LD—principal investigator and study designer. She approved the final version to be published. TL—substantial contributions to the design of the work and was involved in the analysis and interpretation of the data for the work and in revising the work critically for intellectual content. He approved the final version to be published. PR—substantial contributions to the design of the work and was significantly involved in the revising the work critically for important intellectual content. She approved the final version to be published. All the authors agree to be accountable for all aspects of the work in ensuring that questions related to the accuracy or integrity of any part of the work are appropriately investigated and resolved.

## Ethics Statement

This study is a retrospective chart review of infants admitted to the Neonatal Intensive Care Unit at the University of Louisville Hospital. The study was approved by the IRB at the University of Louisville.

## Conflict of Interest Statement

Research was conducted in the absence of any commercial of financial relationships that could be construed as a potential conflict of interest.
